# Efficient Dual-Site Carbon Monoxide Electro-Catalysts via Interfacial Nano-Engineering

**DOI:** 10.1038/srep33127

**Published:** 2016-09-21

**Authors:** Zhen Liu, Zhongyuan Huang, Feifei Cheng, Zhanhu Guo, Guangdi Wang, Xu Chen, Zhe Wang

**Affiliations:** 1 Department of Chemistry, Xavier University of Louisiana, New Orleans, LA 70125, USA; 2 Department of Physics & Engineering, Frostburg State University, Frostburg, MD 21532-2303, USA; 3 Department of Materials Science and Engineering, University of Maryland, College Park, MD 20742, USA; 4 State Key Laboratory of Chemical Resource Engineering, Beijing University of Chemical Technology, Beijing 100029, China; 5 Integrated Composites Laboratory (ICL), Department of Chemical & Biomolecular Engineering, University of Tennessee, Knoxville, TN 37996, USA

## Abstract

Durable, highly efficient, and economic sound electrocatalysts for CO electrooxidation (COE) are the emerging key for wide variety of energy solutions, especially fuel cells and rechargeable metal−air batteries. Herein, we report the novel system of nickel−aluminum double layered hydroxide (NiAl-LDH) nanoplates on carbon nanotubes (CNTs) network. The formulation of such complexes system was to be induced through the assistance of gold nanoparticles in order to form dual-metal active sites so as to create a extended Au/NiO two phase zone. Bis (trifluoromethylsulfonyl)imide (NTf_2_) anion of ionic liquid electrolyte was selected to enhance the CO/O_2_ adsorption and to facilitate electro-catalyzed oxidation of Ni (OH)_2_ to NiOOH by increasing the electrophilicity of catalytic interface. The resulting neutral catalytic system exhibited ultra-high electrocatalytic activity and stability for CO electrooxidation than commercial and other reported precious metal catalysts. The turnover frequency (TOF) of the LDH-Au/CNTs COE catalyst was much higher than the previous reported other similar electrocatalysts, even close to the activity of solid-gas chemical catalysts at high temperature. Moreover, in the long-term durability testing, the negligible variation of current density remains exsisting after 1000 electrochemistry cycles.

Advanced electrocatalysts can power sustainable and efficient fuel cells, metal-air batteries, and electrolyzers. Major advances have been made arising from the emerging field of “surface electrocatalysis” in terms of not only electrocatalysis, but also energy conversion. Low-temperature CO electrooxidation (COE) is one of the most significant processes that critical for the large scale production and storage of natural gas in the chemical form. However, CO catalytic oxidation proceeds at the gas-solid interface through a multi-step kinetically sluggish electron transfer, which may result in deteriorating CO electrocatalysis, and so forth. Effective and sustainable electrocatalysts can reduce the over-potential, expedite the reaction, and maintain the stability, all of which can largely boost the energy conversion efficiency. Traditional Pt/Ru-based alloys in acidic solution have been used as alternatives to more active and CO tolerant electrocatalysts^1^. However, the CO tolerance and activity is still insufficient for commercialization.

The heterogeneous catalysts with enhanced activities at high temperatures have been recently studied due to the development of multicomponent active sites, particularly Au-metal hydroxide and oxide interfaces. In this condition, dual-metal catalytic sites with Au adjacent to oxide have shown higher catalytic activity for CO oxidation via two pathways, (1) increasing the perimeter of the boundary area for gas adsorption, and (2) forming higher activity metal complex with designed metal/ligands combination^2^,^3^,^4^,^5^,^6^. However, most high activity catalysis systems are in solid/gas-phase chemical system at high temperatures. Promoted electrocatalyst/electrolyte system at room temperature have been rarely reported.

Herein, we successfully developed Au nanoparticles bonded nickel−aluminum layered double hydroxide (NiAl-LDH)/carbon nanotubes (CNTs) composite catalysts in ionic liquid. The α- nickel hydroxide nanoplates anchored with Au nanoparticles are expected to create large and high-active boundary area of Au/metal oxide to enhance the CO/oxygen adsorption on the CNTs interconnected conductive networks. Bis (trifluoromethylsulfonyl)imide (NTf_2_) contained in ionic liquid (1-butyl-3-methylpyridinium bis (trifluoromethylsulfonyl)imide, C_4_mpyNTf_2_) was rendered as an additional ligand to metal oxide/hydroxide to stabilize the electrophilic conformation and intermedia in this electrooxidation process. Though the Ni-based oxide/hydroxide composite catalysts have previously been made for COE^7^,^8^, this was the first time that Au assisted crystalline NiAl-LDH was synthesized electrochemically to obtain highly active electrocatalysis for COE in ionic liquid, even close to that of the most active solid/gas-phase chemical at high temperature.

## Results

**Figure 1:**
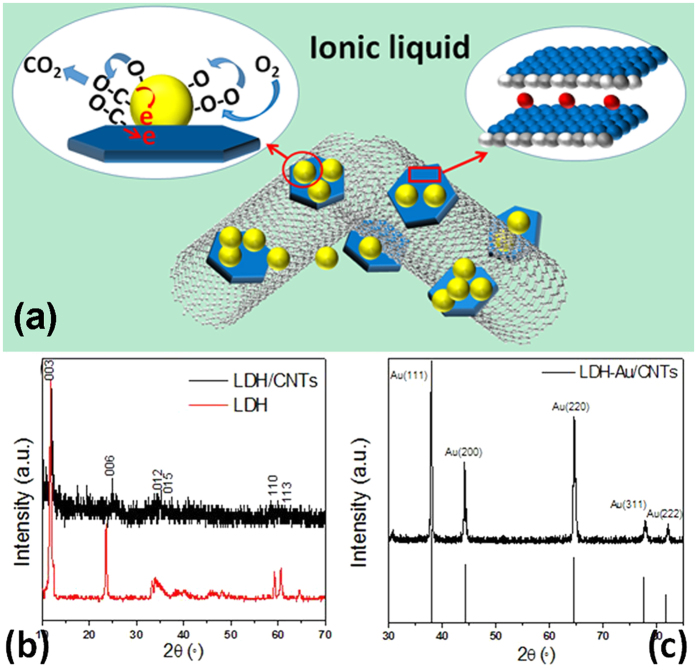
(**a**) Schematic showing the hybrid architecture, LDH crystal structure and COE reaction on the interface, (**b**) XRD patterns of NiAl-LDH/CNTs (black) and NiAl-LDH (red), and (**c**) XRD pattern of NiAl-LDH-Au/CNTs.

Due to its inert nature in the bulk state, gold is generally disregarded in terms of catalytic applications. However, highly dispersed Au on different supports is known to exhibit a surprisingly high activity for several systems in both liquid and gas phases. Since the discovery of remarkable activity in supported gold nanoparticles for CO oxidation^9^, a variety of methods to prepare high activity gold catalysts have been developed, which also lead to several potential applications^10^. However, Au nanoparticles are considered to facilitate the CO adsorption and there is no evidence to indicate their participation in the CO oxidation as an intermediate. For CO oxidation reaction, the mechanism underlying oxygen adsorption and activation for this oxidation reaction remain highly controversial^11^. It is believed that the oxygen adsorption occurs at the metal or the metal support interface. The vacancy sites should then present at the metal-oxide interface as a consequence of the Schottky junction^12^,^13^,^14^,^15^. For the active Ni-oxide catalyst, the structure transformation from NiO to NiOOH was found in the layered Ni(OH)_2_/NiOOH^16^,^17^. It is in direct parallel with the increased catalytic activity. However, it should be noted that only few Ni complexes have been developed for CO conversion due to the strong Ni-OH bonds, which often severely limit the activity of Ni-based catalysts in basic solution. The layered double hydroxides (LDHs) have been considered as an attractive candidate for variable catalysis reactions. It could provide an atomic dispersion interface, facilitating other ligands to reach the Ni center besides -OH. However, it would be necessary to acquire a new ligand to thermodynamically increase the activity of metal catalysts. Bis (trifluoromethylsulfonyl)imide (NTf_2_) anions showed ability to stabilize metal based LUMO and enhance the electrophilicity of catalytic interface. Thus, in NTf_2_–based ionic liquid, transition metal complexes behaved high catalytic activity^18^,^19^, and also produced higher oxygen coverage on the Au surface^20^. For this work, NTf_2_ with sufficient coordination ability to weaken the Ni-OH bond without altering the Ni-OH structural integrity was chosen as anion ionic liquid electrolyte for the COE reaction. Additionally, CNTs could construct a 3-D gas permeable structure to overcome the barrier of electron transfer and gas exchange in the multiple layered structures (Fig. 1a).

The X-ray diffraction (XRD) pattern (Fig. 1b) of the resulting catalyst was consistent with the α-phase Ni(OH)_2_. The thickness of nanoplates was about 5 nm according to the width of (003) and (006) diffraction peaks. The mixing of LDH with CNTs leads to the shift of the peaks of (003) and (006) peaks to higher values of 2θ (as shown in Fig. 1b), indicating a decrease in the interplanar distance. Moreover, the observed changes in peak widths in XRD could be attributed to the changes in crystallite size of LDH because composition of CNTs could reduce the agglomeration of LDH plates and grant the LDH better dispersion^21^,^22^,^23^. Figure 1c indicates that the polycrystal Au nanoparticles were well deposited on the hydride complex.

**Figure 2:**
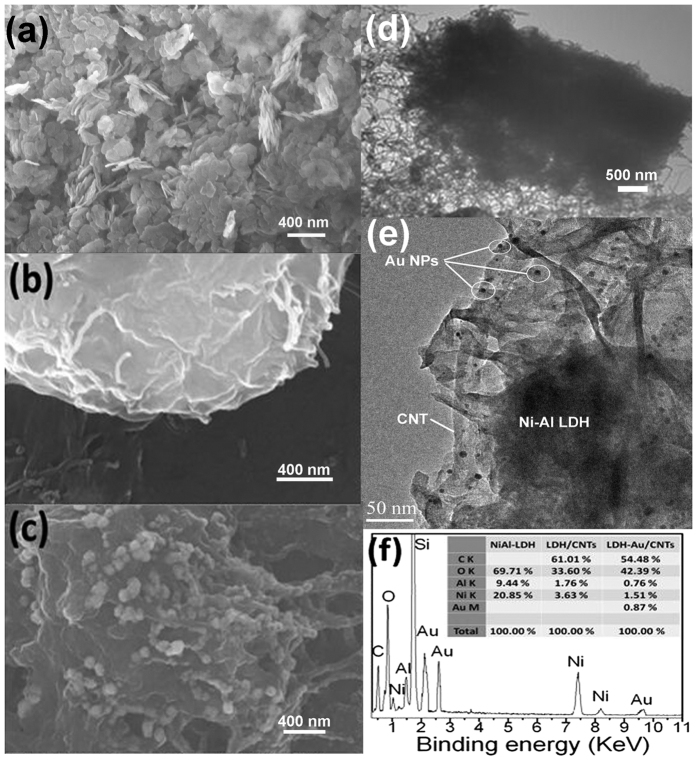
Ultrathin nickel−aluminum layered double hydroxide nanoplates fabricated on carbon nanotubes. SEM images of (**a**) NiAl-LDH nanoplates (**b**) NiAl-LDH nanoplates fabricated over a network of mildly oxidized CNTs and (**c**) Au nanoparticle functionalized NiAl-LDH/CNTs are displayed alongside TEM images of (**d**) NiAl-LDH nanoplates fabricated over a network of mildly oxidized CNTs (**e**) NiAl-LDH nanoplates Au/CNTs and (**f**) EDS analysis result for NiAl-LDH-Au /CNTs hybrid. Insert table lists the element contents of these three materials.

Figure 2 shows the morphology of the fabricated samples. The NiAl-LDH sample features a uniform platelet-like nanoparticulate structure (Fig. 2a). After combining with CNTs, the NiAl-LDH/CNTs sample reveals ultra-thin plates with LDH plate distributed throughout the nanotubes homogeneously. As seen in Fig. 2b, most CNTs were inserted into LDH layers and an interconnected work was built by the CNTs. After the electrochemical deposition, the Au nanoparticles were deposited on the LDH/CNTs surface with a high yield of dispersibility and a diameter of 80–150 nm (Fig. 2c). TEM also displays this formed interconnecting 3D network with the CNTs stacking (Fig. 2e). A porous structure built by CNTs provides a permeable layer for gas exchange. The energy-dispersive spectrum (EDS) results (Fig. 2f) suggest that the molar ratio of Ni/Al remains nearly 2:1 at all times. As shown in Fig. 2 the average size of Au nanoparticles deposited electrochemically is about 5–10 nm and the thickness of LDH plates is several nanometers, which is consistent with the XRD results.

**Figure 3:**
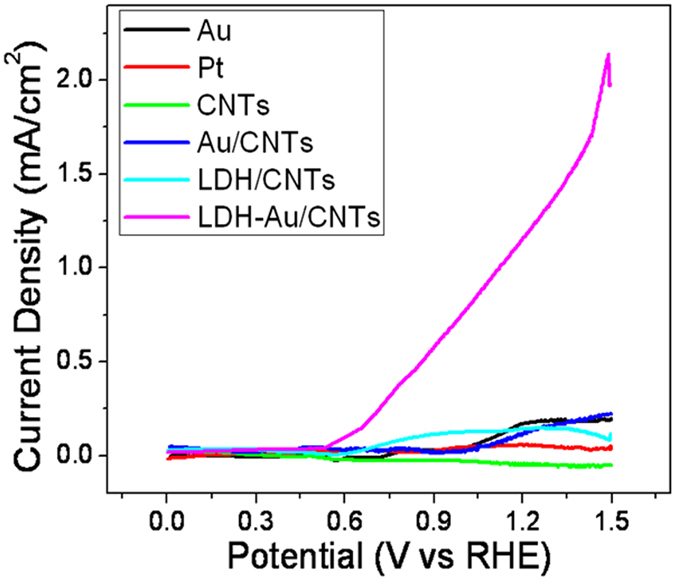
Polarization curves of commercial Au plate and Pt powders, CNTs, LDH-Au/CNTs hybrid and LDH/CNTs catalyst on GC electrode in 0.5 mM KOH in [C_4_mpy][NTf_2_].

The CO electrocatalytic activity of NiAl-LDH/CNTs in ionic liquid (5 mM KOH) was investigated with a standard three-electrode system. The gas permeable membranewas^20^ first uniformly casted (loading ~0.2 mg/cm^2^). The linear sweep voltammograms (LSV) were performed at a high scan rate of 500 mV/s, which is conventional for a ionic liquid electrochemical study^19^. For comparison, a commercial Au plate, Pt powder catalyst, CNTs deposited with Au nanoparticles (with the same ~0.2 mg/cm^2^ loading) was set up for measurement side-by-side. From Fig. 3, the resultant anodic current density for the LDH/CNTs catalysts shows a sharp onset current at ~0.5 V. The presence of LDH (in LDH/CNTs and LDH-Au/CNTs) lowered the onset potential of CO oxidation than pure metal and Au-CNTs (from ~0.7 to ~1.0 V). For LDH-Au/CNTs, a much more negative onset potential and a higher anodic current density were observed in the electrolyte of alkaline IL compared with these in the LDH/CNTs and pure NP Au/CNTs. The TOF value in the electrochemical reactions is calculated from the following equation (1)^24^, which involved Au sites:


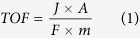


*J* is the current density at overpotential of 0.45 V in A/cm^2^. *A* is the area of the catalysts. *F* is the faraday constant (a value of 96485 *C*/*mol*). *m* is the number of the moles of the active materials (gold) that deposited on the CNTs.

Here, *J* is 2.2 × 10^−3^ A/cm^2^ at 0.45 V, and the loading of the catalysts is about 0.2 mg/cm^2^. Element analysis from XPS (Table S1) show that the mole ratio of Au in the catalysts is 0.5% and the mass ration of Au is 6.28% (Table S1 in Supplementary Information). So the mole ratio of Au in per cm^2^ catalyst is:


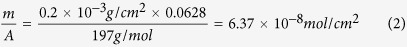



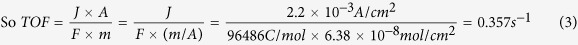


Since some of these sites in the nanoplates were barely electrochemically accessible, this resulted in lower values. Despite this, the measured TOF value in our LDH-Au/CNTs was much higher than those previously reported for mixed Au or Pt nanostructured electrocatalysts and for ionic liquids media^25^,^26^,^27^,^28^,^29^,^30^. Table 1 illustrates the results is close to the reported activity of a high-temperature solid-gas catalysts^31^,^32^,^33^,^34^. As shown in Table 1, Hayden *et al*. synthesized TiO_2_ layers supported Au nanoarticles with different size using physical vapor deposition method. They studied the COE performance of Au/TiOx in HClO_4_ solution and the results showed that Au/TiO_x_ with a gold nanoparticle size of 2.7 nm have a current of 0.1 mA/cm^2^ at 0.6 V (vs RHE). Rodriguez *et al*. described the oxidation of CO in acidic media on Au nanoparticles of 3 and 7 nm on different oxide supports. The gold nanoparticles were deposited on the substrate by using thermos-evaporation in a UHV system from Edwards. They found Au/TiO_2_/HOPG had a current of 0.5 mA/cm^2^ at 0.84 V (vs RHE). In Deng’s work, they prepared Au/CeO_2_ samples by deposition precipitation method with gold particle size of 5 nm. After exposure to H_2_ up to 400 °C, gold diffused out and was in reduced form on the surface.

In this work, the LDH-Au/CNTs shows a much higher current of 2.2 mA/cm^2^ at 0.45 V (vs RHE) indicating that LDH-Au/CNTs has a better catalytic activity for COE than Au/TiO_x_ and Au/TiO_2_/HOPG. The TOF of LDH-Au/CNTs is higher than of Au/CeO_2_ catalyst (0.08 s^−1^). However our method could be performed under natural and room temperature condition.

Here Au nanoparticles play a significant role in the COE process. The most active sites for the CO oxidation have been considered locating at the perimeter of the LDH-Au interface. The oxygen adsorption was enhanced on the Au nanoparticles by the d−π orbital interaction. As a result, the reaction rate increased, thereby the ratio of O_2_/CO adsorption energy^35^,^36^. The most likely process that COE will follow is through a Langmuir-Hinshelwood reaction mechanism on the transition metals^37^,^38^,^39^,^40^. Unlike platinum, Au nanoparticles would provide more sites for active oxygen species while diminishing the CO adsorption. However, due to its low CO surface adsorption rate, Au nanoparticles are not considered as a highly efficient catalyst for the CO oxidation. The boundaries/interface between Au and metal oxide acted as active sites for CO oxidation, thus a maximum activity were achieved by building a high density of Au/oxide boundaries^41^. Similar structures have been considered in many Au-metal oxide catalyst systems^5^,^36^,^42^. In this layered structure, Au nanoparticles were dispersed on the Ni atom layer which then created a large Au/Ni(OH)_2_ interface for the CO oxidation during the electrochemical treatment.

**Figure 4:**
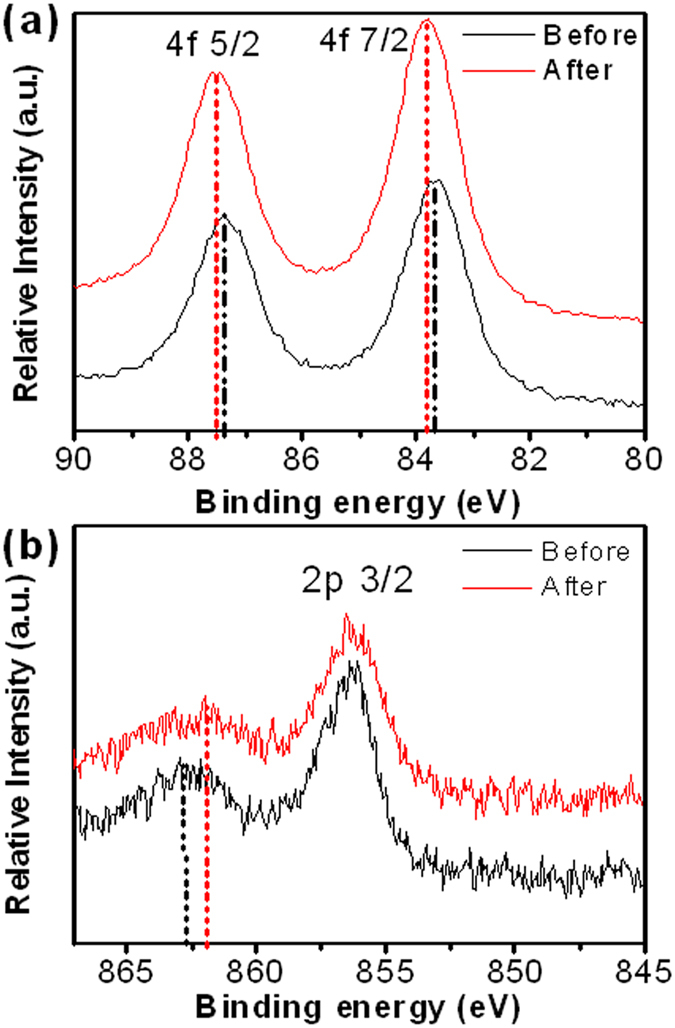
XPS survey spectra before and after CV treatment in ionic liquid, (**a**) high-resolution Au 4f and (**b**) Ni 2p spectra of NiAl-LDH-Au/CNTs hybrid materials.

The behavior of metal catalyst of COE reaction could be characterized by monitoring the metal chemical environmental change during/after CO/O_2_ oxidation^43^,^44^,^45^. As a surface sensitive analysis, X-ray photoelectron spectroscopy (XPS) measurements of metals in presented catalysts were carried out. Figure S1 shows Au 4f of Au/CNTs and LDH-Au/CNTs. The Au binding energies (BE) of Au/CNTs at 4f_5/2_ and 4f_7/2_ are 87.9 eV and 84.2 eV, respectively. This is very close to the metallic Au^46^. Au nanoparticles intrinsically have very close gas adsorption capability with pure Au, thus Au/CNTs showed similar EC behaviors in COE reaction as Au electrode (Fig. 3). While, the negative shifts of Au 4f BE was found in LDH-Au/CNTs. The typical interpretations of these negative shifts of Au 4f BE have been subjected to the electron transfer to the Au, in which Au^*δ*+^ was converted to Au^*δ*− ^47. This charge-transfer was attributed to more absorbed O_2_ species on the Au. The same observation was prior reported in Au particles supported on another Au/metal oxide catalyst as well^48^,^49^,^50^. The increasing of boundary area between the Au and oxides and hydrooxides and partial alloy form could enhance the O_2_ adsorption and catalytical activity sequentially. The interpretations of Au BE as measured by XPS are still often subject to debate with respect to the interactions of Au-support materials, as well as particle size effects^51^. As BE of metallic Au 4f_7/2_ is located at 84.00 eV, any positive shifts (higher BEs) are typically assigned to oxidized state of Au, while these negative shifts of Au 4f BE have been attributed to the electron transfer to the Au^26^,^52^. It was seen in our catalytic system that Au^*δ*+^ was converted to Au^*δ*−^ by a charge-transfer involving O_2_ species instead of the -OH adsorption, as reported^48^,^49^,^50^. The decrease in Au BE from 84.3 to 83.9 eV observed in Au particles supported on Fe_2_O_3_ system is consistent with this prior observations^47^. Therefore, in LDH-Au, the dispersed atomic Ni could construct a very high density of oxide/Au boundaries with Au nanoparticle deposited, likely through a partial alloy form as other Au-metal oxide catalyst. Compared with COE of Au/CNTs, LDH-CNTs had a lower onset potential. Thus, the Ni(OH)_2_ surface may be more critical in the enhanced activity of catalysts. As seen in the Ni 2p plot in Fig. 4b, the appearance of an associated satellite peak around 863 eV indicates the formation of Ni in the LDH/Au structure. It also further confirms the charge-transfer of O_2_. As previously reported^42^, the Au atoms on Ni(OH)_2_ were considered moved via fast surface diffusion during Ni redox in the potential cycling, while Ni atoms was exposed to the alkaline electrolytes for the production of Ni(OH)_2_ on the surface. These atoms are then further attracted to the surface of ligaments via vacancy exchange due to their strong affinity for OH^−^ in the electrochemical system. This proceeds until the surface mixing energy reaches a balance, as seen with the 2p_3/2_ main peaks of Ni-OH at 856.2 eV. A positive shift of Au BE was observed, Fig. 4a, which occurs during and after the CO/O_2_ oxidation. This reveals that the Au surface was covered by atomic oxygen as Au-O formed during the electrochemical treatment. In Fig. 4b, the slight negative shift of Ni 2p_3/2_ satellite peaks clearly indicated the absorbate content change on the Ni surface surrounding Au nanoparticles. This decrease in BE of surface Ni-OH may be attributed to the weakened Ni-OH bonding.

In COE reaction, the major obstacle is adsorption of sufficient oxygen on the surface to form Au-O, since the most of oxygen sources do not thermally dissociate with a high enough rate to deposit significant amounts of oxygen on the surface^53^,^54^. The Au-O barely formed on bare Au in aqueous solution neither on Au/CNTs because of lack of active site, thus there was not significant BE change during COE reaction. Oxygen adsorption represents a major challenge to study reactivity due to the dilemma that most sources of oxygen do not thermally dissociate at a high rate to deposit an adequate amount of oxygen on the surface^53^,^54^. Indeed, the adsorption of O_2_ to yield atomic oxygen is most likely one key step in the oxidation of CO and hydrocarbons^55^. This could be enhanced by increasing the atomic oxygen coverage on the Au surface. Au composition with oxides could enhance electron transfer from the Au to the antibonding states of O_2_, giving rise to ionic bonding between the adsorbed O_2_/Au, and a significant activation of O_2_ towards CO oxidation. Here layer structure with the single atom dispersed metal provided a perfect platform to build this Au/metal oxide boundaries and consequently enhanced following COE. Such atomic oxygen coverage has been observed in gas phase oxidation. However, this is a rare occurrence in aqueous solution since atomic oxygen is unstable in water. Thus, typically the catalytic activity of Au is only possible in gas phase. Here, the ionic liquid provided an inert and high-freedom environment to generate this highly active species.

Usually the high overpotential (rate-determining step) in the aqueous solution that is often seen in metal surfaces is associated with the molecules activation to produce oxidants on the surface^56^. The favorable co-adsorption of CO and hydroxyl species is considered at a lower potential^57^ in alkaline media, where Ni complex is also more favorable. Compared with other ionic liquids, pyrrolidinium bis (trifluoromethylsulfonyl)imide exhibits a lower binding activity to OH^−^ and negligible hydrogen bonding, thereby providing a medium for enhanced OH^−^ adsorption. The rate enhancement at a basic solution suggests that the hydroxyl groups in the solution interact with the gold surface either by facilitating the activation of O_2_ or directly participating in the reaction with CO. Although the role of OH^−^ in the CO oxidation remains unclear, it is proved that the availability of OH^−^ or hydroxyl group greatly accelerated the CO aerobic oxidation rate^58^,^59^. It has recently been shown on water absorbed Pt/Au catalytic surfaces that negatively charged hydroxyl groups were considered as reactive intermediates generated from the “heterolytic dissociation” of water in the CO oxidation. However, the water dissociation and the activation in this process are not energetically favorable^58^,^60^. This is seen that the weakly adsorbed water molecules would not enhance their inherent reactivity^59^. In addition, as the highly active intermediate in the CO oxidation, particularly on the Au surface, the reaction between atomic oxygen and water could also take place. Moreover, the presence of water could make reactions that lead to incomplete products (e.g., –COOH from CO) more thermodynamically and kinetically favorable^59^. Therefore, compared to a general method of subsidizing the system with a hydroxyl group from water, the form of Ni(OH)_2_ in alkaline hydrophobic ionic liquid represented a more efficient pathway to oxidation CO.

**Figure 5:**
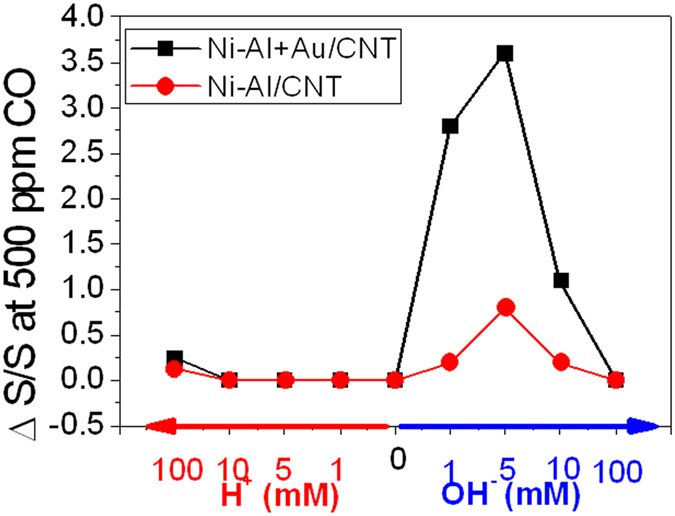
OH^−^ concentration effect towards the current density response of LDH-Au/CNTs catalyst. ΔS/S is the current density change to that without CO.

As reported, the adsorption of OH^−^ could stabilize the CO and O_2_ on the Au^58^,^61^. However, it is possible that excess hydroxyl ions may inhibit the CO oxidation by the irreversible adsorption on the top sites of metal surface, which is considered as the only CO adsorption site^57^. The NiAl-LDH/CNTs and NiAl-LDH-Au/CNTs hybrid material, relative responses of anodic current densities towards 500 ppm CO were presented in Fig. 5 in order to demonstrate the electrocatalytic activity under different OH^−^ concentrations. The greatest electrocatalytic performance was observed at 5 mM OH^−^ in the IL. The requirement of OH^−^ for CO oxidation in ionic liquid is much lower than that in aqueous solution. This allows the reaction to be performed at near neutral environment. 5 mM of OH^−^ is also lower that in alkali promoted Au catalyst for CO gas phase oxidation, in which more than 85% content of catalyst is strongly basic salts^61^,^62^.

**Figure 6:**
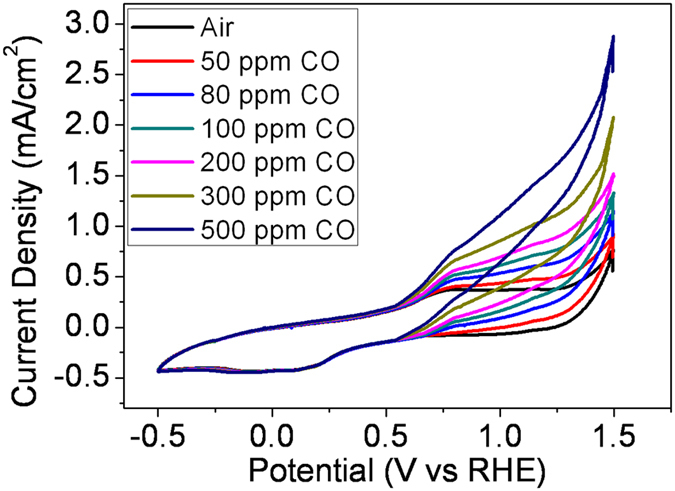
Cyclic voltammograms (scan rate = 500 mV s^–1^) of LDH-Au/CNTs catalyst for the COE reaction in different CO concentration in air.

Cyclic voltammograms were recorded in the presence and absence of CO in air in order to investigate the CO electrocatalytic process of the LDH-Au/CNTs electrodes. For the Ni catalyst, the NiOOH was presumed as common intermedia in the EC aerobic reaction, especially hydrocarbon and CO^63^,^64^,^65^,^66^,^67^,^68^,^69^. The detailed mechanism is still in investigation. Figure 6 shows the recorded cyclic voltammograms in 5 mM KOH in [C_4_mpy][NTf_2_]. Without CO, a pair of quasi-reversible redox peaks of NiOOH/Ni(OH)_2_ were found at 0.2 ~ 0.8 V, black line in Fig. 6. The redox process of Ni(OH)_2_ was proposed to proceed by Equation (4)^70^ as the underlying electrocatalytic oxidation mechanism of the LDH:


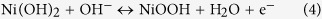


Ni(OH)_2_ was oxidized at a potential of 0.75 V to NiOOH, and then in following cathodic scan it was electrochemically reduced back to Ni(OH)_2_ at 0.2 V. The addition of CO showed an obvious anodic current density increasing, which is originally reported as a CO electrooxidation process by Fleischmann^70^. The NiOOH transformed from Ni(OH)_2_ acted as the direct electrocatalyst for CO oxidation in alkaline media, as CO was oxidized exactly at an onset potential where NiOOH coincides was formed coincidentally. However, the reduction current density of NiOOH remained constant in the negative scan, and there was no consumption of NiOOH observed. Though the specific reaction of the CO oxidation on the surface is considered complex, likely involving multiple steps^71^,^72^. Here, the NiOOH might act simply as an intermediate to trigger the CO oxidation at a low potential. The recorded cyclic voltammograms of LDH-Au/CNTs showed a dramatic enhancement of anodic current density with the addition of only 500 ppm CO in air, indicating a strong catalytic enhancement of the COE reaction. This oxidation is a diffusion controlled process as current linearly with CO concentration (Fig. 6).

**Figure 7:**
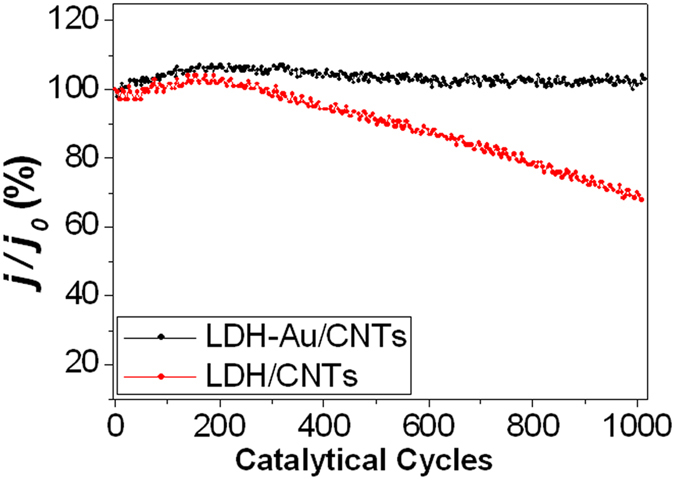
The durability testing of LDH-Au/CNTs catalyst over 1000 catalytic cycles at 500 ppm CO concentration in air.

The variation of current density of COE during 1000 cycles during a long term CV measurement was used to determine the electrochemical stability of both LDH/CNTs and LDH-Au/CNTs (Fig. 7). The current density output of CO oxidation on the LDH-Au/CNTs electrode is more constant than that on the LDH/CNTs. The anodic current density of LDH-Au/CNTs electrode shows only minor decays after 1000 cycles, while the LDH/CNTs electrode exhibits a more significant decrease in current density of CO oxidation. It indicates that the LDH-Au/CNTs electrode is more stable and poisoning-tolerant electrocatalyst of COE in our system. Ni and Ni oxide surface have strong both O_2_ and CO adsorption^73^,^74^. For the active Ni-oxide catalyst, the structure transformation from NiO to NiOOH was found in the layered Ni(OH)_2_/NiOOH. NiOOH was considered as common intermedia of Ni catalyst for the oxygen evolution reduction. Compared with other M^III^ used in LDH, Al hydroxide has high electrochemical stability and high CO tolerance as well. Thus Al could provide solid and robust layer structure of LDH. Additionally, CNTs could construct a 3-D gas permeable structure to overcome the barrier of electron transfer and gas exchange in the multiple layered structures. As combination, LDH-Au could enhance CO oxidation efficiency by containing the large high density of oxide/Au boundaries and CO adsorption, while and high CO tolerant with a stable structure as well.

The activity degradation of COE in alkaline medium is ascribed to two reasons. One is the disintegration of LDH structure, which is mainly associated with the continuous conversion of oxides formation during the long-term potential scanning. Another one is the metal poison accompanied by oxygen/H_2_O evolution reaction on metal catalysts surface^75^,^76^,^77^. For this catalyst, the nickel hydroxide particles of LDH were smaller and more uniform, allowing a tight interaction with Au and CNTs. And this interaction is enhanced to perturb the carbon atoms in the carbonyl groups of CNTs via the formation of M-O-C (M=Ni, Al) bonds^24^. The Au nanoparticle could drastically enhance this effect by facilitating the charge transfer. Furthermore, compared with other LDH complex, alumina structure in NiAl-LDH is more stable, avoiding the structural collapsing during the potential sweeping. And the oxygen/H_2_O evolution was minimized by using the hydrophobic ionic liquids electrolyte. Thus this catalytical system represented an extreme high long-term stability of COE and CO tolerance.

## Conclusions

In this study, we devised and built a high active and stable catalytic interface by combining dual-catalytic sites nanocatalyst with hydrophobic ionic liquid electrolyte. The high active NiAl-LDH nanoplates bonded with Au nanoparticles were constructed on mildly oxidized CNTs and covered by pure C_4_mpyNTf_2_. The resulting catalytical system exhibits higher activity and stability towards COE reaction. The TOF value was measured much higher than those previously reported and no clear current density decay during the 1000 potential cycles under high concentration CO. It is believed that the catalytic species used for triggering COE reaction were NiOOH formed on the electrode surface. And the 5 mM of hydroxide anion provided the more favorite environment for this COE reaction. We further observed that the underlying Au nanoparticles enhanced oxygen adsorption and afforded larger boundary area, facilitating the high CO oxidation activity of the LDH-Au/CNTs complex. These findings led to an electrocatalyst that outperformed in both activity and stability in near neutral solutions, opening a new venue to advanced, low-cost CO conversion electrocatalysts for energy applications.

## Methods

The carbon nanotubes (CNTs, Shenzhen Nanotech Port Co. Ltd, 5–15 μm lengths and 40–60 nm diameters) were treated with 5.0 M nitric acid for 12 h before CNTs were washed with deionized water to neutralize and dried. All other reagents used were analytical grade. After the modified CNTs were sonicated in 50 mL mixture of 0.4 M NaOH solution and 0.1 M Na_2_CO_3_ for 60 mins, 50 mL Ni(NO_3_)_2_ (0.10 M) and Al(NO_3_)_3_ (0.05 M) solution was added into CNTs suspension and the whole solution was adjusted to pH = 10.5. The solid precipitate was collected as LDH/CNTs. 1 mg of the resultant material was dispersed with 100 mL 1.0% Nafion under sonication and pasted on support substrates for using. The electrodeposition of Au nanoparticles was performed from a solution consisting of 1 mM KAuCl_4_ and 0.1 M HClO_4_ by 10 cycles of cycle voltammetry from −0.9 to ~0.1 V. The electrolyte solution was deoxygenated prior to each measurement with pure nitrogen. Au/CNTs was prepared using the same method with LDH-Au/CNTs except the presence of LDH and LDH/CNTs was prepared using the same method with LDH-Au/CNTs except deposition of Au.

A three-electrode electrochemical cell containing the working electrode, a Pt wire counter electrode and a Pt reference electrode; and the relative calibration with ferrocene was described in former work^18^,^19^,^78^,^79^. A glass carbon electrode with surface area of 0.07065 cm^2^ were used as working electrode, and the mass loading of catalysts on working electrode is about 0.2 mg/cm^2^. The mass loading of the catalyst compared to the surface area of electrode was considered to quantify the amount of catalysts in order to evaluate the catalyst properties. The charging current arises when the electrode area, electrode potential, or interface capacitance vary with time. The double layer charging current decays exponentially, according to the cell time constant RuCd, where Ru and Cd are the uncompensated solution resistance and double layer capacitance, respectively. For ionic liquid system, it was shown that the double layer capacitor of an IL was potential-dependent, and also subject to the scan direction of the DC potential. A hysteresis effect in the potential-dependent double layer capacitance at the ionic liquid/metal interface was formed as being due to the slow processes of ions orientation at the interface that take place on a time scale of minutes to hours. And this effect more obvious at low scan rate^80^,^81^,^82^. Thus, by using higher scan rate this time-depended effect could eliminated and the unexpected capacitor response could be avoided in quantification of faradic current. In this complex system, the double layer charging current at the scan rate used (500 mV s^−1^) is similar in value at the IL interfaces under a pure N_2_ atmosphere. So this scan rate could reduce the error caused by the different electrode interface. So scan rate of 500 mV s^−1^ was applied in current work.

The CO oxidation in IL was studied by firstly bubbling CO/air into the IL of interest for 40 mins. The completion of being degassed from each IL was then verified by performing cycle voltammetry with a wide potential window containing both the CO oxidation and oxygen reduction^20^. The powder XRD results were collected on a Shimadzu XRD-6000 diffractometer with Cu Kα radiation (40 kV, 30 mA, and λ = 0.154 nm). The SEM images and Energy dispersive X-ray spectroscopy (EDS) elemental analysis were obtained using a Zeiss Supra 55 scanning electron microscope. TEM characterization was carried out on an H-800 transmission electron microscope. X-ray photoelectron spectroscopy (XPS) measurement was performed on a Thermo Escalab 250 XPS spectrometer using monochromatic Al Kα X-radiation, which was operated in the constant-pass energy mode. The working pressure in the analysis chamber was typically 1 × 10^−7^ Torr. The binding energy scale was calibrated by measuring the C1s peak at 284.8 eV and the accuracy of the measure was ±0.1 eV. Analyses of the Ni 2p, envelopes were carried out at pass energies of 10/20 eV, an energy step size of 0.05 eV, at energy ranges of 895–845 eV. It is consisted with references^52^,^83^.

## Additional Information

**How to cite this article**: Liu, Z. *et al*. Efficient Dual-Site Carbon Monoxide Electro-Catalysts via Interfacial Nano-Engineering. *Sci. Rep.* **6**, 33127; doi: 10.1038/srep33127 (2016).

## Supplementary Material

Supplementary Information

## Figures and Tables

**Table 1:** CO oxidation activities of some benchmark catalysts.

Catalyst	Electrolyte/Interfacea)	Over-Potential	Current Density (mA/cm^2^)	TOF (s^−1^)	Reference
Au/TiOx	HClO_4_/H_2_O	0.6 V (vs RHE)	0.1		25
Au/TiO_2_/HOPG	HClO_4_/H_2_O	0.84 V (vs RHE)	0.5		26
Pt	[Dema] [TfO]	1.5 V (vs RHE)	1.5		27
Pt	[Dema] [TfO]	1.2 V (vs Ag/AgCl)	0.2 (RT)		28
Pt	[PiP_14_] [Ntf_2_]	2.0 V (vs Pt)	0.45		29
Pt	H_2_SO_4_/H_2_O	0.95 V (RHE)	2.5		30
Pt black	KOH/[C_4_mpy] [NTf_2_]	1.0 V (vs RHE)	0.17	1.7 × 10^−4^	This work
Au plate	KOH/[C_4_mpy] [NTf_2_]	1.0 V (vs RHE)	0.1	9.4 × 10^−5^	This work
LDH/CNTs	KOH/[C_4_mpy] [NTf_2_]	0.8 V (vs RHE)	0.2	6.5 × 10^−6^	This work
Au/CNTs	KOH/[C_4_mpy] [NTf_2_]	1.0 V (vs RHE)	0.3	3.03 × 10^−4^	This work
LDH-Au/CNTs	KOH/[C_4_mpy] [NTf_2_]	0.45 V (vs RHE)	2.2	0.357	This work
Au/TiO_2_	Solid/Gas			0.02	31
Au/Al_2_O_3_	Solid/Gas			0.01	31
Au/TiO_2_	Solid/Gas			1.5	32
Au/CeO_2_	Solid/Gas			0.08	33
Co_3_O_4_ nanocubes	Solid/Gas			2.7 × 10^–3^	34

^a)^Electrolyte is for COE reaction and Interface is for solid-gas catalyst.
