# Supplementary material for: Efficient Dual-Site Carbon Monoxide Electro-Catalysts via Interfacial Nano-Engineering
Source: Sci Rep. 2016 Sep 21;6:33127. doi: 10.1038/srep33127 (PMC5030650; doi:10.1038/srep33127)
Supplement: Supplementary Information [file srep33127-s1.doc]

**Supporting Information**

**Efficient Dual-Site Carbon Monoxide Electro-Catalysts via Interfacial Nano-Engineering**

Zhen Liu^1,3,5†^, Zhongyuan Huang^1†^, Feifei Cheng^2^, Zhanhu Guo^4^*, Guangdi Wang^1^**,** Xu Chen^2^*, Zhe Wang^1^*

**^1^**Department of Chemistry, Xavier University of Louisiana, New Orleans, LA 70125, USA.

**^2^**State Key Laboratory of Chemical Resource Engineering, Beijing University of Chemical Technology, Beijing 100029, China.

**^3^**Department of Physics & Engineering, Frostburg State University, Frostburg, MD 21532-2303, USA

**^4^**Integrated Composites Laboratory (ICL), Department of Chemical & Biomolecular Engineering, University of Tennessee, Knoxville, TN 37996, USA.

**^5^**Department of Materials Science and Engineering, University of Maryland, College Park, MD 20742, USA.

^*^Corresponding authors: Z. W. (email: [zwang@xula.edu](mailto:zwang@xula.edu)), X.C. (email: Chenxu@mail.buct.edu.cn) and Z. G. (email: [zguo10@utk.edu](mailto:zguo10@utk.edu)).

^†^These authors contributed equally to this work.





Figure S1 Au 4f of Au/CNT and LDH-Au/CNT

Table S1 The Element mole ratio and mass ratio in LDH-Au/CNTs according the XPS result.

| Element | C1s | O1s | Al2p | Ni2p | Au4f |
| --- | --- | --- | --- | --- | --- |
| Mole ratio | 71.63% | 23.16% | 1.21% | 3.51% | 0.50% |
| Mass ratio | 54.84% | 23.64% | 2.08% | 13.14% | 6.28% |
